# Supplementary figures and images for: Single-cell analysis of isoform switching and transposable element expression during preimplantation embryonic development
Source: PLoS Biol. 2024 Feb 16;22(2):e3002505. doi: 10.1371/journal.pbio.3002505 (PMC10903961; doi:10.1371/journal.pbio.3002505)

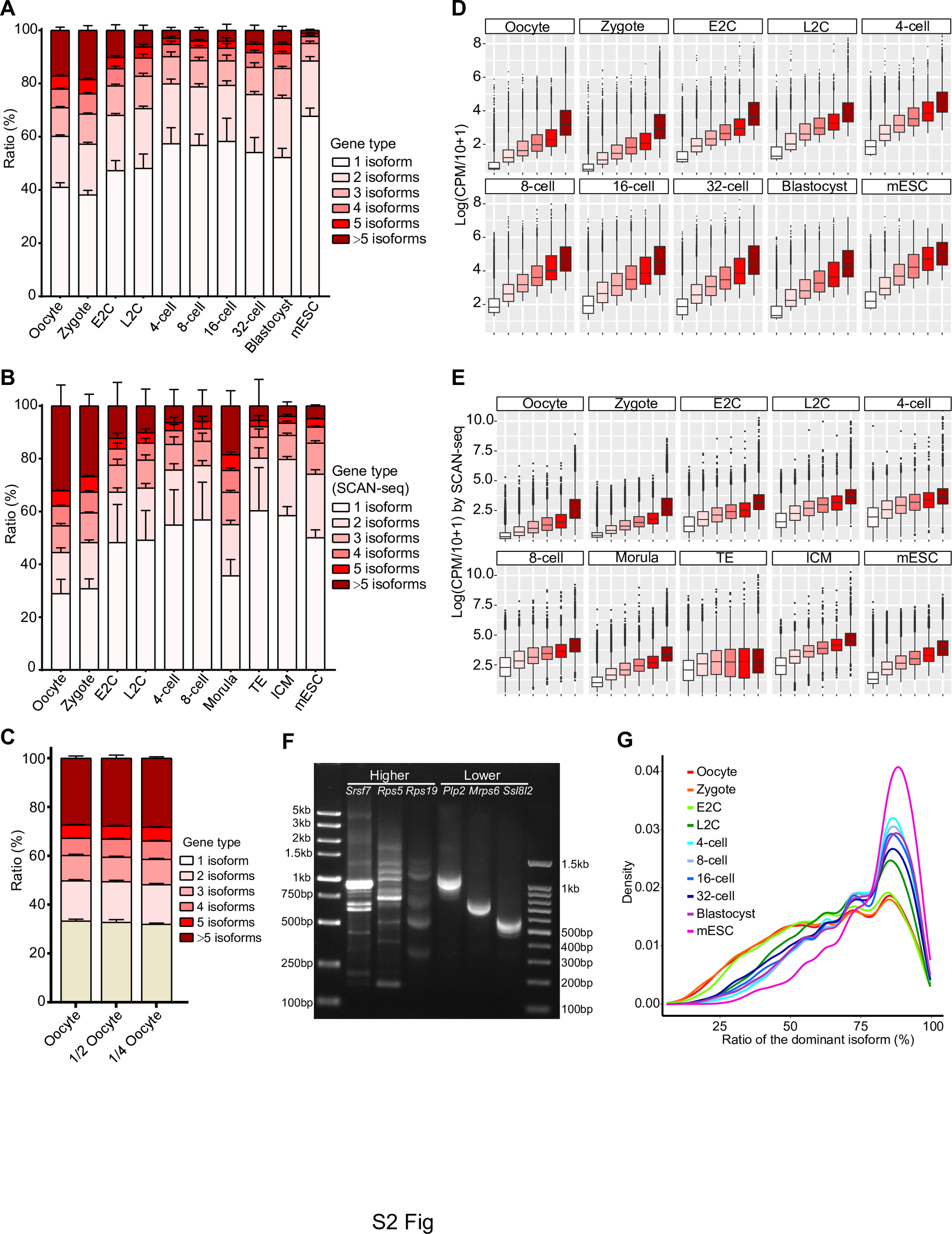

Supplement: S2 Fig — (A, B) The ratios of genes detected with different numbers of isoform types for each stage of mouse embryos and mESCs in this study (A) and SCAN-seq data (B). The raw data for these 2 plots are supplied in S6 Data. (C) The ratios of genes detected with different numbers of isoform types for full oocyte, 1/2 oocyte, and 1/4 oocyte. The raw data for this plot is supplied in S2 Data. (D, E) Expression levels of genes detected with different numbers of isoform types for each stage of mouse embryos and mESCs in this study (D) and SCAN-seq data (E). The raw data for these 2 plots are supplied in S6 Data. (F) Gel view of cDNA amplification products of each gene. Srsf7, Rps5, and Rps19 are examples of highly expressed genes (CPM >100) and Plp2, Mrps6, and Ssl8l2 are lowly expressed genes (CPM <10). The raw image for this plot is supplied in S1 Raw Images. (G) Density plot showing the proportion of the major isoforms in genes expressing multiple isoform types. Only genes detected with UMI counts over 5 were included. The raw data for this plot is supplied in S6 Data. (TIF) [file pbio.3002505.s002.tif]
