# Supplementary material for: Single-cell analysis of isoform switching and transposable element expression during preimplantation embryonic development
Source: PLoS Biol. 2024 Feb 16;22(2):e3002505. doi: 10.1371/journal.pbio.3002505 (PMC10903961; doi:10.1371/journal.pbio.3002505)
Supplement: S1 Raw Images — (PDF) [file pbio.3002505.s010.pdf]

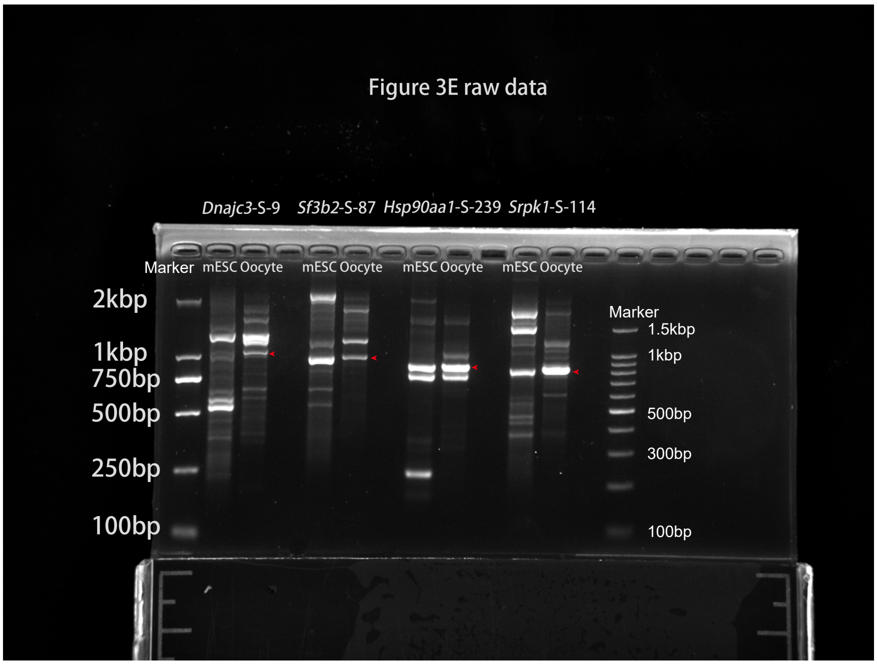

All the samples were amplfied for 32cycles with gene primers, then 8ul of the PCR products were loaded on the lanes respectively. After 45 minutes running under 120V, the Gel was snapped in the UV gel imager machine.

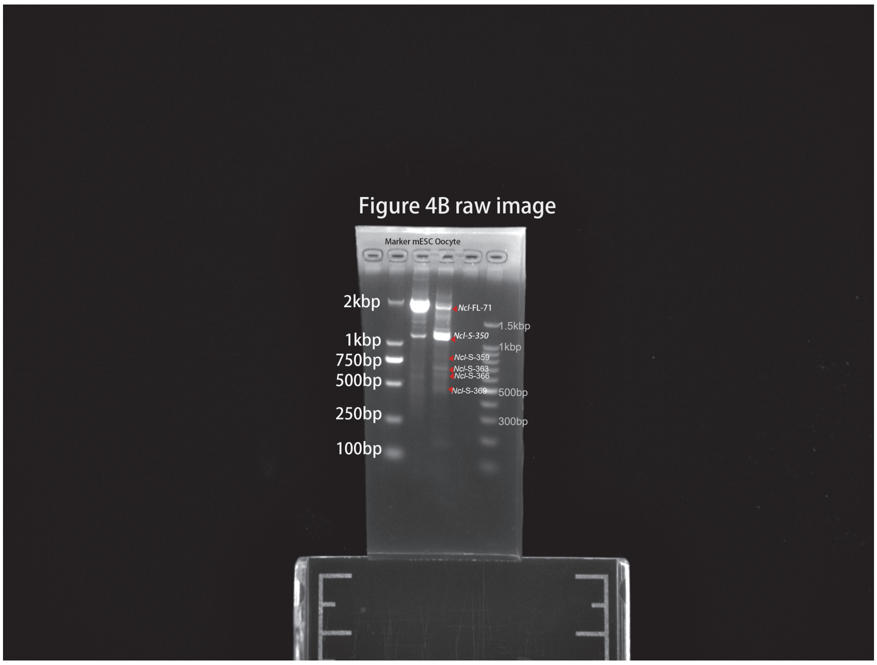

All the samples were amplfied for 32cycles with gene primers, then 8ul of the PCR products were loaded on the lanes respectively. After 30 minutes running under 120V, the Gel was snapped in the UV gel imager machine.

S2F Figure

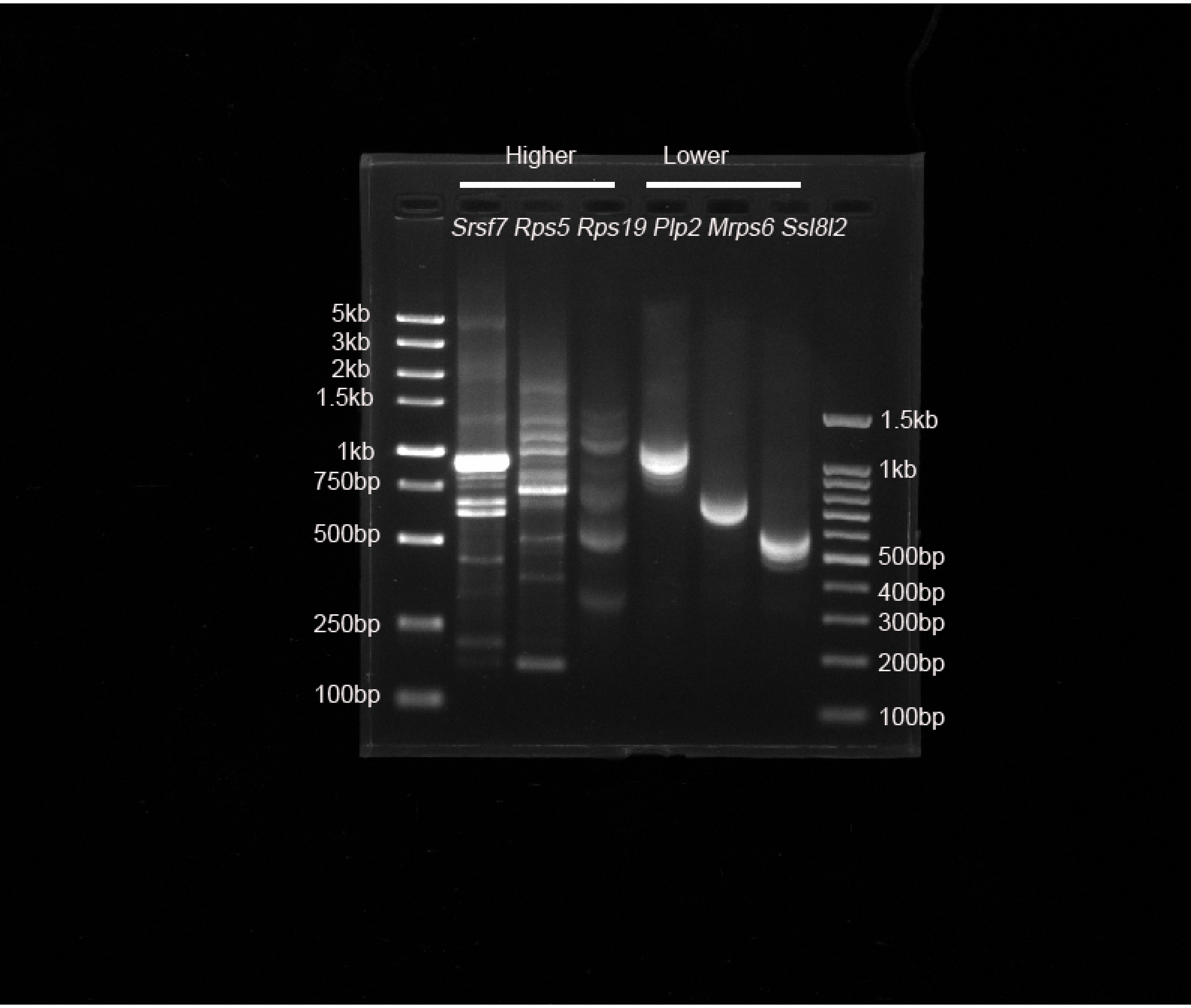

All the samples were amplfied for 32cycles with gene primers, then 8ul of the PCR products were loaded on the lanes respectively. After 50 minutes running under 120V, the Gel was snapped in the UV gel imager machine.
