# Supplementary material for: Single-cell analysis of isoform switching and transposable element expression during preimplantation embryonic development
Source: PLoS Biol. 2024 Feb 16;22(2):e3002505. doi: 10.1371/journal.pbio.3002505 (PMC10903961; doi:10.1371/journal.pbio.3002505)
Supplement: Attachment — Submitted filename: Response to Reviewers-2023-10-20.docx [file pbio.3002505.s019.docx]

**Reviewer #1**

Wang, Shi et al present a study to observe full length transcripts using nanopore sequencing in murine preimplantation embryos on a single cell level. This technique aids the identification of transcript isoforms as well as transposable elements. The authors conclude on a switch in isoform diversity and dynamics, which coincides with zygotic genome activation (ZGA). Additionally, the technique allows for the observation of transposable elements (TEs) on a family as well as locus specific level. Although the authors present an interesting adaptation of the SMART-seq2 technique for low input samples, the manuscript seems to lack several key analyses and controls, as well as biological interpretation of their findings as described in more detail below. Additionally, several minor comments are listed below to improve the clarity of the text.

Author Response: We really appreciate your suggestions and have added experimental results to validate isoform diversity for different genes and analytical results relevant to TE chimeric transcripts. More biological interpretation of isoform and TE expression are discussed in the revised manuscript.

**Major comments**

1. In line 182-184 the authors state that genes expressing more isoforms are among highly expressed genes. However, this could be caused by a sampling artifact considering that at this sequence depth it is more likely to detect more isoforms of genes that are highly transcribed compared to genes that are expressed at lower levels. The authors should confirm the lower isoform diversity of lowly expressed genes by comparing RT-PCR and sanger sequencing results of high and low expressed genes as was done for other findings in their study.

Author Response: We appreciate your consideration that the higher isoform diversity of highly expressed genes could possibly be caused by the relatively shallow sequencing depths. To prove the hypothesis of lower isoform diversity of lowly expressed genes, we randomly chose 3 highly expressed genes (CPM>100) and 3 lowly expressed genes (CPM<10) in mESC to confirm their isoform diversity by RT-PCR. *Srsf7* (CPM=105), *Rps5* (CPM=236) and *Rps19* (CPM=371) were chosen and designed a primer that can amplify out 13, 5, 6 isoforms respectively according to our RNA-seq data. *Plp2* (CPM=4.1), *Mrps6* (CPM=7.7) and *Ss18l2* (CPM=7.0) were chosen and each only had 1 isoform according to our RNA-seq data. As shown in Figure R1 below, there appeared more isoform types in the gel than we detected by sequencing. So it is true that we could not capture all the isoform types of each gene, possibly due to the sequencing depth. Nevertheless, the highly expressed genes are validated to express more types of isoforms than the lowly expressed genes. We have added the results in the revised manuscript.

Figure R1. Gel view of cDNA amplification products of each gene. *Srsf7*, *Rps5*, *Rps19* represent highly expressed genes (CPM>100) and *Plp2, Mrps6, Ssl8l2* represent lowly expressed genes (CPM<10).

2. In line 209-213, the authors discuss isoform candidates that are truncated at the 3' end. They state (as a biological finding) that there are no isoforms detected without poly (A) tail. This conclusion is in my view, incorrect, considering that the technique used (Smart-seq2) uses a poly-A-based RT approach and thus requires the transcript to have a poly(A) tail (as a consequence, SMART-seq-2 cannot naturally detect transcripts without poly(A) tails).

Author Response: We agree with your comment that only transcripts with poly(A) tails could be captured and detected using our method. We intended to state that the 3-prime partial transcripts were truly exist and sanger sequencing proved they contained poly(A) tails. Thus this kind of transcripts could be captured by our methods. The finding here was that the 3-prime partial transcripts did not contain stop codons. As the original sentence was easy giving rise to misunderstandings, we modified the statement as “Meanwhile, no stop codons were identified in these transcripts according to the sanger sequencing results”.

3. Along these lines, the authors claim that isoforms are much more frequently truncated at the 5' end. Can the authors confirm that this is indeed a biological finding and not instead a result of incomplete reverse transcription?

Author Response: Thank you for your considerate advice. To check whether the 5-prime partial transcripts were real or just a result of incomplete reverse transcription, we referred to the mouse TSS database[1] and calculated the ratio of transcripts containing a known TSS within each transcript type. As shown in Figure R2 below, the 5-prime partial transcripts contained relatively lower ratio of transcripts overlapping with annotated TSS, indicating these transcripts were more likely being generated from incomplete reverse transcription. Because we could not specify which of these transcripts were artificial, we did not make further investigations on them, even though they showed higher abundances than the 3-prime partial transcripts. We added the results in the revised manuscript.

Figure R2. Ratio of the transcripts overlapped with annotated TSS. Transcripts with the 5-terminal locating within 200bp of the CAGE peaks are regarded as overlapped transcripts.

4. In figure 4B a DNA gel is shown accompanied by the statement that shorter isoforms are more abundant than the full-length transcript. Although this may very well be the case, a DNA gel can be hard to interpret for this purpose as longer fragments will appear brighter than shorter fragments due to the nucleotide staining. The authors should consider confirming this finding by bioanalyzer or by other more quantitative techniques.

Author Response: We appreciate your suggestion and reanalyzed the PCR products with Qsep100. As shown in Figure R3, the short isoforms of NCL is more abundant that the full-length one in mouse oocyte, but not the mESC. We have updated the gel picture with mESC control and added the quantification result by bioanalyzer in the revised Figure 4B.

Figure R3. (A) Gel picture of *Ncl* RT-PCR products from mESC and mouse oocyte. (B) Q-sep result showing the RT-PCR product of *Ncl* transcripts from mouse oocyte.

5. It is a novel and interesting finding that DNA transposons are found in this dataset to be expressed to a similar degree as LINEs, SINEs and LTRs. However, this finding does not get explained or highlighted by the authors. This should be added to the discussion.

Author Response: We sincerely appreciate the reviewer's perceptive observation. In fact, the total expression level of DNA transposons in our dataset was also far less than the retrotransposons (Figure R4A, R4C). However, the locus expression shown that DNA transposons were comparable to LINEs, SINEs and LTRs from the active loci, suggesting much fewer DNA transposon locus are active (Figure R4B). We believe this is an advantage of our method, offering more detailed information on TE regulation patterns. To avoid misunderstanding, we also modified Fig. 5A to show the UMI count of each TE super-family in revised manuscript (Also shown in Figure R4C).

Figure R4. (A) Total expression level of each TE super-family. (B) Expression level of each expressed TE locus belong to different super-families. (C) Total UMI counts of each TE super-family in single cells.

6. According to the methods, for the TE analysis, only transcripts fully mapped to the TE sequence are taken into consideration. However, several studies (among which a recent study by Modzelewski, Cell 2021) have identified transcript isoforms where TEs are used as an alternative promoter for gene expression. It would be highly relevant for the authors to investigate chimeric transcripts containing both genic and TE regions in their dataset.

Author Response: We acknowledge your advice and searched for the TE chimeric gene transcripts. Considering that most existing software is primarily developed for pair-end short-read data (for example ChimeraTE ([2])) and is not suitable for long-read data, we conducted this analysis in our study by developing our own analysis scripts (See the method section in manuscript). In total, we identified 6143 TE chimeric transcripts counting for 7609 UMIs from all the cells. Over 80% of the transcripts were detected with only one copy in only one cell. One of the reason could be low expression levels of these transcripts. Considering limited sequencing depth from the TGS, it is hard to detect these transcripts. Another reason might be the current bioinformatical methods, which are not very suitable for analyzing the TE chimeric transcripts. As the recently study by Berrens et.al ([3]) also captured quite limited number of TE-derived isoforms in each cell (~200 for the mouse 2-cell sample and ~20 for human iPSCs). Increase of TGS throughput and the development of bioinformatics would help us to further explore on such interesting regulations. We have added the results (S4 Table) and discussions in the revised manuscript.

**Minor comments**

1. The text should be thoroughly checked for spelling and grammar mistakes for clarity and correctness. Several examples of spelling errors (incomplete list);

a. Line 74; "Calss1" should be "class I".

b. Line 85; "Lous" should be "locus".

c. Figure 5b + legend "Expressed TE locus" should be "Expressed TE loci".

d. In the methods in line 419 the authors state that expression values are normalized by copy number per 100,000 mapped reads. However, CPM is typically per 1,000,000 mapped reads.

Author Response: Thanks for bringing these errors to our attention. We have meticulously reviewed the entire manuscript and addressed all instances of spelling mistakes and improper words. In the methods part of CPM calculation, as there were averagely less than 100,000 reads for each cell due to limited sequencing depth of TGS, we normalized the total reads to 100,000/cell, showing as CPM/10. All the CPMs in the article and figures have been replaced by CPM/10.

2. Line 77 introduces MERVL and MT2_Mm. It should be made clear the MT2_Mm is the LTR of the internal (originally coding) sequence of MERVL for a better understanding of the author's statement.

Author Response: We really appreciate your considerate suggestion and we added the introductions to the specific TE subfamilies we highlighted in the results.

3. On several occasions the text is currently missing references. For example;

a. Line 82-85; there are several reviews that discuss this problem and computational tools/wet lab techniques that address this problem that should be cited here.

b. Line 132 after the statement that the mouse oocyte/zygote contains more RNA molecules compared to later stages.

c. Line 181 a reference is missing for this public (?) SCAN-seq dataset and method.

d. Line 286; there are several reviews that have highlighted this particular challenge of TE biology that can be cited (e.g. Lanciano and Cristofari, Nature Reviews Genetics 2020)

Author Response: Thanks for the valuable suggestion. We have added the related references accordingly.

4. All figure legends and labels should be improved for clarity. In particular;

a. Figure 2 lacks information on how the rows in heatmap D-E are ordered within each cluster.

b. Figure 5a lacks information on what the y-axis reflects (actual raw counts? Normalized counts? Number of TEs that were identified or number of counts that are inside TEs?).

c. Figure 5C legends also does not contain information on whether the dots represent individual blastomeres or TE loci.

d. The legend of Figure 5D states that unique loci are mapped (n=3894), but this number would make more sense if the heatmap shows unique TE families (e.g. MT2_Mm). Is this correct?

e. Similar to figure 2, it is unclear how the rows of the heatmaps in figure 5 (5D, 5G and 5H) are ordered/clustered. This should be mentioned in the figure legend. Additionally, it should be highlighted in the legend that Figure 5G-H only show examples of individual loci and does not reflect all insertions of a specific family.

Author Response: Thank you for the considerate suggestions. We have modified the figure legends accordingly. The TEs (raws) in Figure 5D were ordered by their averaged expression levels within each stage. The y-axis in Fig. 5A represent the UMI counts of each TE superfamily in individual cells. Each dot in Fig. 5C represent one cell. For Fig. 5D, we added the color bar and also provided the corresponded TE information in S3 Table, where readers can find the exact TE subfamily information for each locus. We have added the instructions in the legend. In Fig. 5G and 5H, we only exhibited highly expressed TE loci (with mean expression >1 log count across all cells).

5. Line 303-304; The statement "Our data shows that Lx7 …" should either be toned down or moved to the discussion as this is a very strong statement without experimental proof for support.

Author Response: We appreciate your suggestion and toned down the statement as “In our data, Lx7 emerged as the most active LINE1 subfamily since L2C stage (Fig 5F, S3D Fig).” and removed the sentence “But the exact LINE1 elements playing such a role is unclear.”

6. Line 352; "On the other hand, abnormal expression of TE is harmful to human"; it should be specified that this is the case for most differentiated tissues, not during embryo development.

Author Response: Thank you for the precise advice. We have modified the sentence as “On the other hand, abnormal expression of TE in most differentiated tissues is harmful to human”.

**Reviewer #2 (Jong Kyoung Kim, signs review)**

Summary: In this manuscript, the authors developed a protocol for long-read single-cell RNA sequencing to quantify the expression levels of isoforms and locus-specific transposable elements in single cells. The protocol, modified from SCAN-seq (using nanopore sequencing) to use PacBio HiFi sequencing, was applied to mouse oocytes, zygotes and blastomeres. They characterized the dynamic isoform switching patterns and locus-specific expression of transposable elements (TEs) during mouse embryonic development.

The present manuscript is potentially important because it provides a valuable single-cell resource for understanding the complete landscape of gene expression during early embryonic development by profiling individual isoforms and locus-specific TEs. However, there are several major points that should be addressed to substantiate the authors' claims.

Author Response: We deeply appreciate the valuable comments and constructive suggestions offered. The Reviewer's reviewing efforts are highly regarded, and we share the view regarding the substantial contribution of our study in revealing individual isoform expression and locus-specific TE expression during early embryonic development. With respect to the Reviewer's feedback, we have carefully revised the manuscript, resulting in significant enhancements. Please refer to the comprehensive point-by-point response below for detailed insights into the revisions made.

**Major points:**

1. The authors used SIRV spike-ins to evaluate the accuracy of quantifying isoform expression levels. For the SIRV spike-ins, they should cite the bioRxiv preprint by Paul et al., (<https://doi.org/10.1101/080747)>. Fig. 1D does not provide any information on the accuracy of measuring isoform expression levels. The authors should provide this information by comparing the true and estimated isoform expression levels per cell, and by showing the relationship between technical noise (squared CV) and the mean isoform expression levels. I'm also wondering if the accuracy and technical noise of quantifying isoform expression in single cells are affected by the total amount of mRNAs in the cells. This can be done by evaluating the accuracy and technical noise of isoform quantification for each developmental stage, since the total amount of mRNAs per cell would decrease from oocytes to blastomeres.

Author Response: Thanks for your kind reminding and we have added the citation related to SIRV spike-ins. In our analyses, ERCC, which contains 92 types of RNA, each is of different amount when mixed. This allowed us to create a fit curve to estimate how the detected amount of the RNAs matched the true amount. But for SIRV, all the isoforms are equally pooled. So we could only decide whether they are corrected mapped and determined in Fig. 4D.

To evaluate how the total amount of mRNAs affect ERCC quantification, we calculated the correlation of detected ERCC amount in different cells within each stage. Lower correlation represents less accuracy and higher technical noise in measuring ERCC amount. As shown in Figure R5，the correlation value generally increased from oocyte to blastocyst. This indicate that RNAs of lower amount in a cell (the oocyte and zygote contain the most abundant RNAs in each cell, thus ERCC is relative less in these samples) were less accurately detected. This is a common issue in single-cell sequencing and could hopefully improve through two ways: 1. increasing sequencing depth, which eliminate the randomness of detecting the lowly expressed genes; 2. optimizing single-cell RNA amplification reaction to ensure capturing and amplifying of as many cellular RNAs as possible.

Figure R5. Correlation between detected UMI counts and absolute spiked abundances of ERCC genes in each cell at different stages.

2. Fig. S1A and B: Since the number of isoform types is strongly influenced by the expression levels of genes (Fig. S1C and D), the rich isoform diversity observed in early mouse embryos may be an artefact by their increased total amount of mRNAs per cell. The authors should show that technical replicates of a cell from early embryos with reduced total amount of mRNAs have similar isoform diversity. The technical replicates of a cell can be obtained by splitting a cell lysate into multiple aliquots. The same also applies to Fig. 3C.

Author Response: Thanks for your insightful comments and suggestions. To validate whether the rich isoform diversity observed in early mouse embryos was truly a biological finding or just the result of increased total amount of mRNAs per cell, we collected mouse oocytes and split one cell into 2 and 4 aliquots, respectively. The result showed that ratios of genes containing different number of isoform types are almost consistent among intact oocytes, 1/2 oocytes and 1/4 oocytes, suggesting that the detected isoform diversity was hardly affected by the amount of mRNAs (Figure R6A). Thus the rich isoform diversity observed in early mouse embryos was probably a true biological finding instead of an artefact.

In addition, we also checked whether the efficiency of detecting different types of transcripts was affected by the total amount of mRNAs per cell. Fig. 3C showed the absolute amount of the 3-prime partial transcripts and it is reasonable that the whole cells are detected with more abundant transcripts than only partial of the cells. We intended to tell that the relative amount of the 3-prime partial transcripts was more enriched in the maternal RNA content, as Fig. 3B shows. To further specify the higher ratio of the 3-prime partial transcripts in mouse oocytes and zygotes was not caused by more starting mRNAs in single cells at these stages, we also calculated the ratios of different transcript types in split oocyte samples. As shown in Figure R6B, there is no significant difference between the ratios of the 3-prime partial transcripts in 1/2 oocyte, 1/4 oocyte and the intact oocyte, suggesting that this type of transcript was indeed more abundant in the maternal RNA content. We have added the new results in the revised manuscript.

Figure R6. (A) Ratios of genes expressed different number of isoforms in full oocyte, 1/2 oocyte and 1/4 oocyte. (B) Ratios of different types of transcripts in full oocyte, 1/2 oocyte and 1/4 oocyte. The red box shows the statistic test of ratios of the 3-prme partial transcripts between each pair of sample group.

3. A list of genes showing dynamic isoform switching during early embryonic development should be provided as a supplementary table, and their shared biological properties should be systematically characterized and discussed.

Author Response: Thanks for the valuable suggestion. We understand the significance of providing a gene list that showed isoform switch during preimplantation embryonic development. To address this, we have added the detailed information in a new sheet of Supplementary Tables S1. Besides, we calculated the number of isoform switch events happened between each adjacent stages. Importantly, zygote-E2C and E2C-L2C, during which the minor and major ZGA events happen respectively, were detected with more events (Figure R7A, B). GO analysis showed that these genes were related to translation, cell division, etc. (Figure R7C). The results suggested that isoform switch might be an important regulation mechanism in zygotic gene activation. We have added the results in the revised manuscript.

Figure R7. (A) Number of isoform switch events between each adjacent embryonic stages. (B) Number of genes that have isoform switch events between each adjacent embryonic stages. (C) GO analysis of genes showed isoform switch during preimplantation embryo development.

4. Fig. 5G and H: I'm wondering if neighboring genes of TE copies with similar expression patterns show more correlated expression during embryonic development. The role of TEs as putative regulators should be further characterized.

Author Response: Thanks for your insightful suggestions. To specify whether these TEs are putative regulators during preimplatation embryo development, we calculated the correlations between expression levels of the stage specific TEs (in original Fig. 5G, 5H) with their neighboring genes. Meanwhile, non-TE neighboring genes were also randomly extracted as controls. The results show that the TE neighboring genes are significantly positively correlated with corresponding TEs in expression (Figure R8A). Examples are also shown in Figure R8B (also shown in revised Fig. 5G), exhibiting that the expression pattern of genes located nearby the ERVL and LINE1 is similar to the TE themselves. Therefore, the TEs, especially ERVL and LINE1, may play a role in stage-specific gene expression regulation. We have added the results in the revised manuscript.

Figure R8. (A) Correlation values between expression levels of representative stage-specific LINE and ERVL loci (UMI counts>5 in each subfamily) and their adjacent genes (<10kb distance). Non-adjacent genes are defined as genes over 1Mb away from the TE loci. (B) Heatmap showing examples of consistent expressions between specific TE loci and their adjacent genes along developmental stages.

5. The discussion section seems truncated and abruptly ended. The limitations of the proposed protocol and the current study should be discussed.

Author Response: Thank you for valuable suggestion. We added discussions on the limitations of the current study in the revised manuscript. Please refer to the details in Line 379-398 and Line 429-440.

Minor comments:

1. The terms "3’ prime" and "5’ prime" through the manuscript and figures should be replaced with "3'" (or "3-prime") and "5'" (or "5-prime"), respectively.

Author Response: Thank you for your suggestion and we have replaced the spells throughout the manuscript and figures.

2. Many typos in the manuscript (e.g. "down regulated" in line 156, "showes" in line 241, and "is consists of" in line 305)

Author Response: Thanks for bringing these errors to our attention. We have meticulously reviewed the entire manuscript and addressed all instances of spelling mistakes and improper words.

3. Line 278-279: The sentence is not matched with Fig. 5C.

Author Response: We are sorry for the unclear statements and we have changed the sentences as “In both calculation ways, the oocyte and zygote were detected with higher expression levels. However, more different expression patterns were observed between total TE super-family expression and individual TE locus expression. For example, LINE is slightly downregulated from E2C to 32-cell stage when looking at all the super-family, but transcription level at each locus are upregulated.”

**Reviewer #3**

In this manuscript, the authors present a novel approach utilizing HIT-scISOseq, a hifi long-read sequencing technique from PacBio, to investigate isoform switching during embryo development in mouse preimplantation embryos. The authors have previously published a method called SCAN-seq (Fan X et al. PLoS Biol. 2020) for long-read single-cell RNA sequencing in mouse preimplantation embryos. In this study, they aim to enhance transcript abundance estimation by incorporating unique molecular identifiers (UMIs) and spike-in correction and evaluate the capability of the modified platform to identify isoforms.

The sequencing component of this study introduces two noteworthy advancements. Firstly, the authors successfully adapt the HIT-scISOseq method for low-throughput applications, enabling the quantification and identification of isoforms associated with not only alternative splicing but transposable elements. Secondly, they classify transcripts into various types, such as 3-prime partial, 5-prime partial, complete, and insertion, and perform quantitative analyses to assess changes in these categories.

However, it remains challenging to discern the specific benefits of this approach in revealing or supporting biological themes related to embryo development. Consequently, I recommend some revisions to enhance the impact and citation potential of this paper.

Author Response: We deeply appreciate your valuable feedback and constructive suggestions offered. We have followed your advice, adding analyses to reveal the rationale of our single-cell experiments and to better understand the isoforms. The point-to-point response is shown in below.

**Major points**

1. The number of blastomeres used in this study appears to be limited, with only 161 cells analyzed from various stages of preimplantation embryos. This sample size may not provide sufficient statistical power to discern cellular differences within each stage. Additionally, the rationale behind utilizing single-cell RNA sequencing instead of bulk-long read sequencing technology is not clearly explained. It would be beneficial to clarify the necessity of a single-cell-based approach in this study. In particular, the selection of only 13 blastocyst cells raises concerns about their representativeness, as these cells appear to be more clustered compared to cells from other stages (as shown in Figure 2B and 2C).

Author Response: We really appreciate your concerns about the sample size and the rationale of using single-cell RNA-seq instead of whole embryo sequencing. Due to limited sequencing throughput by TGS and we aimed to achieve higher sensitivity for each single cell, only 161 cells were sequenced from eight stages of preimplantation embryos. Using these samples, we identified stage specific gene, isoform and TE expressions, offering new information and perspectives regarding molecular regulation of early embryonic development. We agree with your idea that more samples could enhance our findings, and we have added this limitations of our work in the revised discussion.

Single-cell-based approach not only offered us differences across developmental stages, but also the cell heterogeneity within a specific stage. Using the low throughput method, we marked cells from the same embryo during the experiment, thus we can further evaluate fate differentiation of the blastomeres within an embryo. As shown in Figure R9A and R9B, the two blastomeres in 2-cell embryo showed high consistence on both gene and isoform expression level, and became more different to each other from 4-cell stage, suggesting fate divarication could be revealed at 4-cell stage on transcription level. When comparing the heterogeneity between cells from different embryos of the same stage, the correlation values also decreased along with developmental stages. While the decreased cell-cell correlation at zygote stage probably resulted from batch effects of different embryo replicates. We believe this result fully demonstrated the advances of single-cell approach instead of bulk analyses in the current work and we have added to the revised manuscript.

Figure R9. (A) Correlation coefficients of blastomeres within the same embryos or different embryos at the same stage base on gene expression data. (B) Correlation coefficients of blastomeres within the same embryos or different embryos at the same stage base on isoform expression data. (C) PCA plot of embryonic cells from Fan et al., 2020. The box indicates the blastocyst cells. (D) PCA plot of embryonic cells from Wang et al., 2021. The box indicates the blastocyst cells.

In the PCA plot in Fig. 2B and 2C, since we put all cells together, the main differences were from different stages, resulting in relative central distribution of cells from the same stages. This phenomenon was also observe in previous studies, such as SCAN-seq and scNOMeRe-seq (Figure R9C and R9D)([4, 5]). All these datasets showed relatively gathered distribution of blastocyst cells. As there is differentiation of multiple cell fates in blastocyst (including ICM, TE, PE), the cell-cell heterogeneity is much larger than earlier stages. With the low throughput methods, we aimed to investigate intra-embryo heterogeneity. On the other hand, it is difficult to collect enough cell samples from the blastocyst stage. We believe future studies using high throughput approaches are needed to fully specify the transcript regulations in cell fate specification at blastocyst stage. We add the discussions in the revised manuscript.

2. Isoforms can play distinct roles based on various factors such as changes in expression levels, structural conformation, loss of enzymatic activity, and rewiring of protein interactions. To provide a more comprehensive understanding of isoform dynamics and their implications in reprogramming during development, the authors should conduct further analyses. One approach could involve leveraging protein residue-level function annotation tools, such as FunFam from Christine A. Orengo, to identify functional dissimilarities among isoforms. By employing these tools, the authors may gain insights into the specific isoforms driving the developmental stages of preimplantation embryos. Integrating such analyses into the study would enhance our understanding of the functional implications of isoform switching during embryo development.

Author Response: We appreciate your suggestions that use functional annotation tools to identify functional dissimilarities among isoforms. We tried FunFam (http://www.cathdb.info/search/by_sequence) with the isoform switch examples and 3-prime partial isoforms, including *Ncl*, *Caprin2, Csnk2a1, Dnajb6, Stk16*, etc., but no matched structural domains or functional families could be found in the dataset for the novel isoform types. For other genes such as *Cnot7, Cth, Commd6, Nsmce2*, no structural or functional differences were observed between each pair of isoforms, even though they showed differences in the amino acid sequences. We further expanded the analysis list to all the genes showing isoform switch during preimplantation embryo development. Finally, 7 genes were identified with functional/structural changes when isoform switch happened. As shown in Figure R10 below.

Figure R10. Expression pattern of gene isoforms which showed switch during preimplantaion embryo development. The exact functional/structural domains predicted in each isoform are annotated based on the CATH database (http://cathdb.info/search/by_sequence).

3. An important consideration is that even when using different embryos, the presence of inserted transposable elements (TEs) should be preserved if these TEs play a crucial role in development. Therefore, the identification of functional TEs can offer an opportunity for lineage tracing across different stages of development. This aspect underscores the significance of employing a single-cell-based approach. By characterizing the expression and activity of TEs at the single-cell level, the authors can potentially unravel their functional roles and trace the lineage of cells across different developmental stages. This approach would provide valuable insights into the impact of TEs on developmental processes, further justifying the use of a single-cell-based approach in this study.

Author Response: We acknowledge your suggestion regarding the existence of functional non-random TE insertion events during development. However, it is important to note that the precise insertion events can only be determined through genome sequencing, as per our current understanding. In terms of transcriptome analysis, we can infer potential functional TEs by identifying stage-specific TE elements that exhibit consistent expression across cells from biological replicates. However, it is crucial to recognize that TE expression itself is dynamic and changes with developmental stages. While we can observe the expression of TEs in different cells, we cannot determine which cells belong to the same lineage. For instance, if TE at site A is expressed in progenitor cells, it is possible that this TE is silenced in differentiated cells. Hence, we cannot definitively conclude that differentiated cells do not originate from progenitor cells based solely on TE expression patterns. Similar to considering each TE locus as an independent gene, we can infer which cells have similar gene expression profiles, typically indicating cells of the same type. However, currently, there is no method to solely infer lineage relationships between cells based on functional gene expression. Lineage tracking can be achieved through two approaches: identifying cells of different lineages through cell-specific mutations (such as mitochondrial DNA)[6] or introducing artificial cell barcodes that can be inherited during cell proliferation to identify cells within the same lineage[7-12]. Among these methods, the CRISPR-induced artificial cell barcodes could further accumulate mutations during cell passages to infer the relationships of parent and daughter cells. Algorithms, including LinTIMaT [13] and LineageOT [14], are enabling lineage reconstruction from RNA sequencing data. But these are not fit for TE expression analysis. Consequently, lineage tracing cannot be accomplished solely through TE expression information within our technical system.

So we hope you can reconsider this comment or if you have specific strategies for utilizing single-cell TE expression data for lineage tracing, we would be eager to consider and explore them.

**Minor points**

- Please show loadings when you make PCA plots in figure 2B and 2C.

- Please add more information for figure captions and legends (e.g. -1 to 1 in 2D).

- Typo: Page 10 line 74 calss1 -> class1

Author Response: Thank you for your suggestion and we have modified the manuscript and figures accordingly. We used the 'FactoMineR' R package for conducting PCA analysis. Loading (i.e., standardized coordinates) is not directly provided in the 'FactoMineR' package. Instead, it returns principal coordinates. Therefore, we calculated the loading by dividing the variable coordinates along a dimension by the square root of the eigenvalue of that dimension. More information on this calculation can find at <http://factominer.free.fr/question/FAQ.html>. The PCA loadings for Figure 2B and 2C are supplied in S1 Table.

**References**

1.	Abugessaisa I, Noguchi S, Hasegawa A, Kondo A, Kawaji H, Carninci P, et al. refTSS: A Reference Data Set for Human and Mouse Transcription Start Sites. J Mol Biol. 2019;431(13):2407-22. Epub 2019/05/11. doi: 10.1016/j.jmb.2019.04.045. PubMed PMID: 31075273.

2.	Oliveira DS, Fablet M, Larue A, Vallier A, Carareto CMA, Rebollo R, et al. ChimeraTE: a pipeline to detect chimeric transcripts derived from genes and transposable elements. Nucleic Acids Res. 2023. Epub 2023/08/24. doi: 10.1093/nar/gkad671. PubMed PMID: 37615575.

3.	Berrens RV, Yang A, Laumer CE, Lun ATL, Bieberich F, Law CT, et al. Locus-specific expression of transposable elements in single cells with CELLO-seq. Nat Biotechnol. 2022;40(4):546-54. Epub 2021/11/17. doi: 10.1038/s41587-021-01093-1. PubMed PMID: 34782740.

4.	Fan X, Tang D, Liao Y, Li P, Zhang Y, Wang M, et al. Single-cell RNA-seq analysis of mouse preimplantation embryos by third-generation sequencing. PLoS Biol. 2020;18(12):e3001017. Epub 2020/12/31. doi: 10.1371/journal.pbio.3001017. PubMed PMID: 33378329; PubMed Central PMCID: PMCPMC7773192.

5.	Wang Y, Yuan P, Yan Z, Yang M, Huo Y, Nie Y, et al. Single-cell multiomics sequencing reveals the functional regulatory landscape of early embryos. Nat Commun. 2021;12(1):1247. Epub 2021/02/25. doi: 10.1038/s41467-021-21409-8. PubMed PMID: 33623021; PubMed Central PMCID: PMCPMC7902657.

6.	Ludwig LS, Lareau CA, Ulirsch JC, Christian E, Muus C, Li LH, et al. Lineage Tracing in Humans Enabled by Mitochondrial Mutations and Single-Cell Genomics. Cell. 2019;176(6):1325-39.e22. Epub 2019/03/05. doi: 10.1016/j.cell.2019.01.022. PubMed PMID: 30827679; PubMed Central PMCID: PMCPMC6408267.

7.	Weinreb C, Rodriguez-Fraticelli A, Camargo FD, Klein AM. Lineage tracing on transcriptional landscapes links state to fate during differentiation. Science. 2020;367(6479). Epub 2020/01/25. doi: 10.1126/science.aaw3381. PubMed PMID: 31974159; PubMed Central PMCID: PMCPMC7608074.

8.	Oren Y, Tsabar M, Cuoco MS, Amir-Zilberstein L, Cabanos HF, Hütter JC, et al. Cycling cancer persister cells arise from lineages with distinct programs. Nature. 2021;596(7873):576-82. Epub 2021/08/13. doi: 10.1038/s41586-021-03796-6. PubMed PMID: 34381210; PubMed Central PMCID: PMCPMC9209846.

9.	Raj B, Wagner DE, McKenna A, Pandey S, Klein AM, Shendure J, et al. Simultaneous single-cell profiling of lineages and cell types in the vertebrate brain. Nat Biotechnol. 2018;36(5):442-50. Epub 2018/04/03. doi: 10.1038/nbt.4103. PubMed PMID: 29608178; PubMed Central PMCID: PMCPMC5938111.

10.	Loveless TB, Grotts JH, Schechter MW, Forouzmand E, Carlson CK, Agahi BS, et al. Lineage tracing and analog recording in mammalian cells by single-site DNA writing. Nat Chem Biol. 2021;17(6):739-47. Epub 2021/03/24. doi: 10.1038/s41589-021-00769-8. PubMed PMID: 33753928; PubMed Central PMCID: PMCPMC8891441.

11.	Liu K, Deng S, Ye C, Yao Z, Wang J, Gong H, et al. Mapping single-cell-resolution cell phylogeny reveals cell population dynamics during organ development. Nat Methods. 2021;18(12):1506-14. Epub 2021/12/04. doi: 10.1038/s41592-021-01325-x. PubMed PMID: 34857936.

12.	He Z, Maynard A, Jain A, Gerber T, Petri R, Lin HC, et al. Lineage recording in human cerebral organoids. Nat Methods. 2022;19(1):90-9. Epub 2022/01/01. doi: 10.1038/s41592-021-01344-8. PubMed PMID: 34969984; PubMed Central PMCID: PMCPMC8748197.

13.	Zafar H, Lin C, Bar-Joseph Z. Single-cell lineage tracing by integrating CRISPR-Cas9 mutations with transcriptomic data. Nat Commun. 2020;11(1):3055. Epub 2020/06/18. doi: 10.1038/s41467-020-16821-5. PubMed PMID: 32546686; PubMed Central PMCID: PMCPMC7298005.

14.	Forrow A, Schiebinger G. LineageOT is a unified framework for lineage tracing and trajectory inference. Nat Commun. 2021;12(1):4940. Epub 2021/08/18. doi: 10.1038/s41467-021-25133-1. PubMed PMID: 34400634; PubMed Central PMCID: PMCPMC8367995.
