# Supplementary material for: Single-cell analysis of isoform switching and transposable element expression during preimplantation embryonic development
Source: PLoS Biol. 2024 Feb 16;22(2):e3002505. doi: 10.1371/journal.pbio.3002505 (PMC10903961; doi:10.1371/journal.pbio.3002505)
Supplement: Attachment — Submitted filename: response to reviewers.docx [file pbio.3002505.s020.docx]

Reviewer #1: The authors have added quite some more analyses to TEs (as myself and the other reviewers had suggested), in particular the neighboring gene analysis.

Overall, they addressed the comments I raised fully. However, I again strongly suggest the authors and the editorial team to improve the text for grammar correctness and clarity. Additionally, one minor suggestion is that I think the authors made a mistake in the label of figure 5C. As far as I understand the figure shows TE expression on a locus specific level in the upper panel and on a subfamily level on the lower level, however the legend states the opposite.

Author Response: We glad that you are satisfied with our revisions. Thank you for your careful reviewing. We have corrected the mistake of figure legend in Figure 5C. In addition, we have gone through the full manuscript and improved the text for grammar correctness and clarity.

Reviewer #2 (Jong Kyoung Kim, signs review): The authors have satisfactorily addressed my comments.

Author Response: We glad that you are satisfied with our revisions. Many thanks to your effort in reviewing our work.
